# Effect of Ultrasound Pre-Treatment on Extraction and Characterization of Collagen from Bactrian Camel Skin

**DOI:** 10.3390/polym15081943

**Published:** 2023-04-19

**Authors:** Jing He, Rui Shi, Rimutu Ji

**Affiliations:** 1 College of Food Science and Engineering, Inner Mongolia Agricultural University, Hohhot 010010, China; 2 China-Mongolia Joint Laboratory of Biopolymer Application “One Belt One Road”, Hohhot 010018, China

**Keywords:** *Bactrian camel* skin, collagen, sonication, characterization

## Abstract

The objective of this study was to evaluate the effect of ultrasound pre-treatment on the characterization from Bactrian camel skin. It was possible to produce and characterize collagen extracted from Bactrian camel skin. The results showed that the yield of collagen was higher in ultrasound pre-treatment (UPSC) (41.99%) than the pepsin-soluble collagen extraction (PSC) (26.08%). All extracts were identified as type I collagens using sodium dodecyl sulfate polyacrylamide gel electrophoresis and retained their helical structure, as confirmed through Fourier transform infrared spectroscopy. The scanning electron microscopy analysis of UPSC revealed that some physical changes were caused by sonication. UPSC had smaller particle size than PSC. The viscosity of UPSC always plays a leading role in the range of 0–10 Hz. However, the contribution of elasticity to the solution system of PSC increased in the range of 1–10 Hz. Moreover, ultrasound-treated collagen had superior solubility property at pH 1–4 and at <3% (*w*/*v*) NaCl than non-ultrasound treated collagen. Therefore, the utilization of ultrasound for the extraction of pepsin soluble collagen is a good alternative technology to expand the application at industrial level.

## 1. Introduction

Animal tissues contain abundant levels of collagen, which mainly exists in skin, bone, cartilage, tendon, and other connective tissues, accounting for about 30% of total animal protein [1]. Up to 29 types of collagen are present in animal tissues, with various structures and molecular attributes [2]. In mammals and fish, the most abundant form is type I collagen, whose perfect biocompatibility and controlled biodegradability, has led to its wide use in pharmaceuticals, biomedicines, cosmetics, and food [3].

According to statistics, 89% of camels are dromedaries located in North Africa, West Asia, and Australia, and the other 11% are Bactrian camels, mainly distributed in China and Mongolia [4]. China is one of the main distribution areas of Bactrian camels. There are 405,300 camels in China, of which 42.72% (173,000) are found in Inner Mongolia [5]. Camel skin is the main by-product after camel slaughter, which accounts for 10–15% of the body weight [6]. Camel skin is considered a potential source of collagen.

Generally, collagen fiber-rich tissues, such as skin or tendons, are treated with neutral salts, acids, alkalis, and proteases to extract collagen [7]. Unfortunately, such extraction techniques are frequently lengthy, inefficient, and result in copious collagenous tissue residues during the extraction process. Low-intensity ultrasound is a non-destructive, safe, and efficient auxiliary extraction method. This technique has been widely used to extract collagen and change the protein’s functional and structural properties, and its thermal stability [8,9]. Camel skin can be used as a high-quality raw material for collagen extraction. However, few studies have investigated the properties of its collagen, and have mainly focused on gelatin. Nowadays, the highest yield of camel skin gelatin after alkaline treatment is 22.60% [6,10]. However, these conventional extraction methods are usually time consuming, and a considerable amount of insoluble collagen is left behind. It can be completed in a shorter time with high reproducibility using the ultrasound technology. Moreover, using ultrasound reduces solvent consumption and exhibits higher final product purity [7,11]. This study aimed to study the impact of ultrasound treatment on skin collagen physicochemical and functional properties, and evaluate the feasibility of the ultrasound pre-treatment method in comparison to the chemical method.

## 2. Materials and Methods

### 2.1. Materials

Fresh skin of a three-year-old *Bactrian camel* was purchased from local producers in Alxa League, Inner Mongolia, China. The skin was dissected into 3 × 3 cm^2^ pieces and frozen at −20 °C for 2 weeks. Neutral protease (1398, Longyuan Bioengineering Co., Ltd., Shandong, Jinan, China), pepsin from porcine gastric mucosa (1/10,000, Sigma-Aldrich, St. Louis, MO, USA), and SDS-PAGE gel preparation kit, protein maker, and sodium dodecyl sulfate (SDS) were purchased from solaibao Technology Co., Ltd. (Beijing, China). Other reagents were of analytical grade and obtained from Sinopharm Chemical Reagent Co., Ltd. (Shanghai, China).

### 2.2. Pre-treatment and Sample Preparation

Before use, the *Bactrian camel* skin was thawed at 4 °C and cut into 3 cm × 3 cm blocks, added with 0.5% Na_2_CO_3_ with a skin/solution ratio of 1:10 (g/mL), soaked for 12 h, and then washed with distilled water. To remove the hair on the *Bactrian camel* hides, 0.6% neutral protease with a skin/solution ratio of was added to the soaked raw hides and incubated at 25 °C for 4 h. After depilation, the *Bactrian camel* skin was cut into 0.5 cm × 0.5 cm pieces, soaked in 5% Na_2_CO_3_ solution at 1:10 (g/mL), and incubated with continuous stirring at 20 °C for 18 h to remove fat and pigment. The fat-removed raw materials were soaked in 2% NaCl solution at 1:10 (g/mL) and incubated with continuous stirring at 20 °C for 18 h to remove salt-soluble non-collagen components, and then washed with distilled water. Finally, the pretreated *Bactrian camel* skin was placed at −20 °C until use.

### 2.3. Extraction of Collagen from Pretreated Bactrian camel Skin

#### 2.3.1. Pepsin-Solubilized Collagen (PSC)

All PSC extractions were performed at 4 °C. 0.5 M acetic acid and pretreated skin tissues were mixed at a ratio of 1:20 (skin: acid (*w*/*v*)), together with 4% (*w*/*v*) pepsin and incubated for 48 h. Following centrifugation (centrifuge 5810 R, Eppendorf, Hamburg, Germany) at 10,000 g/min at 4 °C for 20 min, NaCl in 0.05 M Tris buffer was added to the supernatant to a final concentration of 0.9 M. The mixture was centrifuged for 10 min at 10,000 g/min and the pellet was dissolved in 5 volumes of 0.5 M acetic acid. Following sequential dialysis against 0.1 M acetic acid followed by distilled water for 24 h each (with two changes of dialysis solution each day), the sample was lyophilized (PSC from *Bactrian camel* skin).

#### 2.3.2. Ultrasound-Treated Pepsin-Solubilized Collagen (UPSC)

The pretreated *Bactrian camel* skin was suspended in 0.5 M acetic acid containing 4% (*w*/*v*) pepsin at 1:20 (*w*/*v*) ratio and incubated for 24 h at 4 °C. An ultrasonic cell pulverizer (JY88-IIN, Xinzhi Biotechnology Co., Ltd., Ningbo, China) was used to extract PSC ultrasonically. The reaction was carried out at a single frequency of 24 kHz and a power of 200 W. The samples were sonicated for 20 min and then centrifuged for 10 min at 10,000 g/min for 10 min. Subsequent steps were the same as those detailed in Section 2.3.1. The UPSC was lyophilized and placed at −20 °C until further use.

### 2.4. Characterization of Collagen

#### 2.4.1. Yield of Collagen

The hydroxyproline content in collagen was measured according to the method of Li et al. [12]. The yield of PSC and UPSC was calculated and compared using Equation (1).

(1) $$\mathrm{Yield}\;\left(\%\right)=\frac{A1\times B1}{A2\times B2}\times 100\;\%$$

where A1 represents the collagen Hyp content (mg/g); A2 represents the *Bactrian camel* skin Hyp content (mg/g); B1 represents the collagen dry weight (g); and B2 represents the *Bactrian camel* skin dry weight (g).

#### 2.4.2. Amino Acid Composition

The amino acid composition of PSC and UPSC was analyzed using an amino acid analyzer (L8900, Hitachi, Tokyo, Japan) using a previously published method [13].

#### 2.4.3. Sodium Dodecyl Sulfate Polyacrylamide Gel Electrophoresis (SDS-PAGE)

The electrophoretic mobility patterns of collagens were assessed in accordance with Song et al. [14]. Briefly, electrophoretic separation was performed using a 5% stacking gel and an 8% separating gel system, and 5 μL of 0.5 mg/mL collagen solution in 0.5 mol/L acetic acid was separated.

#### 2.4.4. UV Scanning

A UV-visible spectrophotometer (LAMBDA 365, Perkin-Elmer, Waltham, MA, USA) was used to determine the collagens’ UV spectra according to a previously published method with slight modifications [15]. Dry collagen solution (0.5 M in 0.1 M acetic acid) was added to a quartz cell and the UV absorbance spectrum was determined at 200–400 nm.

#### 2.4.5. Fourier Transform Infrared (FTIR) Spectroscopy

An FTIR spectrometer (Nicolet iS10, ThermoScientific, ABD, Waltham, MA, USA) was used to measure the collagen samples’ FITR spectra. A 2 mg collagen sample was mixed with 100 mg of potassium bromide (KBr) and formed into a tablet, which was mounted in the sample holder of the FTIR spectrometer. The samples were scanned and measured using a 500–4000 cm^−1^ spectral range with a 2 cm^−1^ resolution [16].

#### 2.4.6. Zeta Potential and Particle Size distribution

The method of Indriani et al. [16] was used to measure the zeta potential of the collagen samples using a zeta potential analyzer. The zeta potential showing zero at pH was identified as the isoelectric point (pI). A ZEN 3600 laser particle size analyzer (Malvern Instruments, Malvern, UK) was used to measure the particle size of collagen in solution (0.2 mg/mL distilled water shaken for1 h at 25 °C).

#### 2.4.7. Scanning Electron Microscopy (SEM)

A scanning electron microscope (TM4000; Hitachi Ltd., Hitachi, Japan) was used to observe the morphology of lyophilized collagen. Freeze-dried collagen was cut into small pieces and fixed after spraying gold. SEM images were captured under an accelerating voltage of 10 kV at ×200 magnification [12].

### 2.5. Rheological Properties

Rheological characterization was carried out in a rotational rheometer (Thermo RS6000) using a 49 mm diameter parallel stainless steel plate with a 1 mm gap. Collagen cross-linking behavior was determined using dynamic frequency sweeps. In the linear viscoelastic region, the sample was selected with a strain setting of 0.5%, a scanning frequency of 0.01–10 Hz, and a temperature of 25 °C. Dynamic temperature sweeps were performed from 10 to 70 °C over a linear range of 0.5% constant strain, and an accuracy of 1.0 °C/min. We then analyzed changes in the elastic modulus (G′), viscous modulus (G″), and loss factor (tanδ = G″/G′) of collagen under the two modes.

### 2.6. Solubility

Freeze-dried samples of PSCs and UPSCs were dissolved in 0.5 M acetic acid and stirred slowly for 6 h at 4 °C to achieve a concentration of 2.5 mg/mL.

#### 2.6.1. Determining Collagen Solubility at Different pH Values

The collagen solutions were adjusted to achieve a pH range of 1–10. The samples were stirred evenly, centrifuged for 20 min at 10,000 *g* and 4 °C, and the biuret method was used to measure the protein content in the supernatant [14]. The relative solubility was calculated using Equation (2).

(2) $$\mathrm{Relative}\;\mathrm{solubility}\;\%=\frac{W_{\mathrm{pH}}}{W_{\mathrm{pH}.\max}}\times 100\;\%$$

where W_pH_ is the protein content in mg/g at different pH values. W_pH.max_ is the maximum dissolved amount of protein when the pH value changes, mg/g.

#### 2.6.2. Collagen Solubility under Different NaCl Values

The collagen solutions (5 mL) were mixed with 5 mL of acetic acid (0.5 M) with different concentrations of NaCl 1–6% (*w*/*v*) containing final NaCl concentrations of 1–6% (*w*/*v*), after centrifugation at 10,000 g/min for 30 min at 4 °C. Using the above method, the protein content in the supernatant was determined and the relative solubility of collagen was calculated using Equation (3) [14].

(3) $$\mathrm{Relative}\;\mathrm{solubility}\;\%=\frac{W_{\mathrm{NaCl}}}{W_{\mathrm{NaCl}.\max}}\times 100\;\%$$

where W_NaCl_ is the protein content under different NaCl concentrations, mg/g. W_NaCl.max_ is the maximum dissolved amount of protein when the NaCl concentration changes, mg/g.

### 2.7. Statistical Analyses

IBM SPSS software (Version 24.0, IBM Corp., Armonk, NY, USA) was used to carry out all the statistical analyses. The least significant difference (LSD) test was used to compare the means. Statistically significant differences were accepted at *p* < 0.05. In the figures, data are displayed as the mean ± SD.

## 3. Result and Discussion

### 3.1. Yield of Collagen

An appropriate ultrasound treatment time was beneficial to the extraction of collagen. In this experiment, the yields of PSC and UPSC were 26.08 ± 2.26% and 41.99 ± 1.57%, respectively. Ultrasonication increased the collagen content of UPSC by 16% compared with that of PSC. This increase was possibly caused by the cavitation and mechanical effects of ultrasound [17].

### 3.2. Collagen Amino Acid Composition

The amino acid composition is an important factor that determines collagen’s physicochemical properties, including thermal stability, cross-linking ability, and solubility. PSC and UPSC had similar amino acid profiles. As shown in Table 1, in PSC and UPSC, 1/3 of the total amino acid content was glycine. Glycine is the most abundant amino acid in all collagens, being vital in the formation of the superhelical structure [18,19]. All collagens had high levels of proline, glutamine, alanine, and arginine; their methionine, histidine, and tyrosine contents were relatively low; and they lacked tryptophan. Our findings are representative of the amino acid composition of type I collagen [16,20].

### 3.3. Collagen Electrophoretic Mobility Patterns

**Figure 1:**
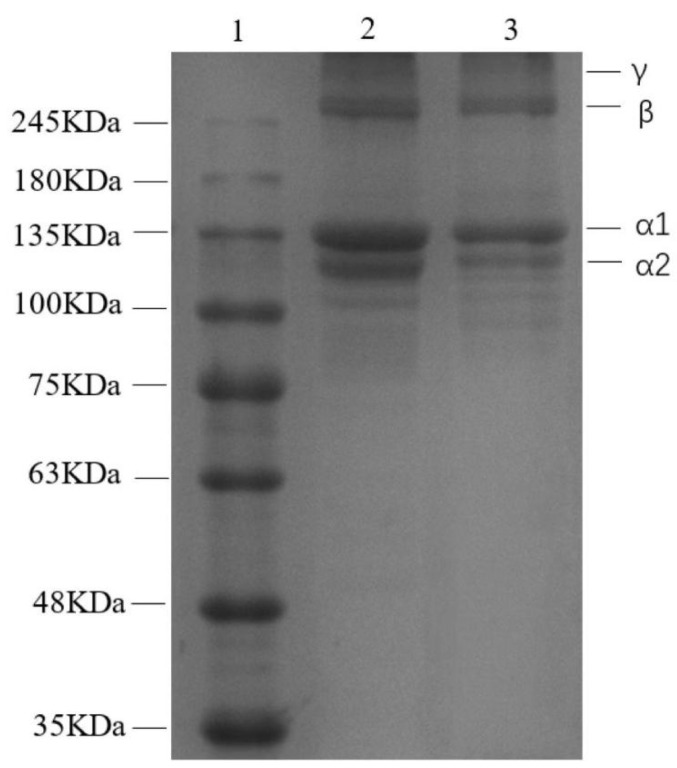
Patterns of all collagens extracted from *Bactrian camel* skin, assessed using sodium dodecyl sulfate polyacrylamide gel electrophoresis (1, Standard protein marker; 2, PSC; 3, UPSC).

PSC and UPSC exhibited similar protein electrophoretic mobility patterns, with α1 and α2 chains and their dimers (β chains) and trimers (γ chains), respectively (Figure 1). Previous studies have confirmed that type I collagen-specific bands ranged from 127 to 150 kDa (α1-and α2-chains) and 200–250 kDa (β-chains), where the α1-chain band intensity was twice that of the α2-chain [21]. The existence of two different α1- and α2-chains confirmed that the collagens of *Bactrian camel* skin belonged to type I collagen. The identification of the γ-chain suggested abundant intermolecular and intramolecular collagen cross-links [22]. In collagens isolated from bovine, porcine, and avian skin, weaker bands (α1, α2, and β) were detected, but no γ-chains were observed [23]. Moreover, in this study, the intensities of the α1- and α2-chains for UPSC were lower than those of PSC. This is believed to be the result of sonication-induced structural changes, especially to the density of the α-chain [24].

### 3.4. Collagen UV Absorption Spectra

**Figure 2:**
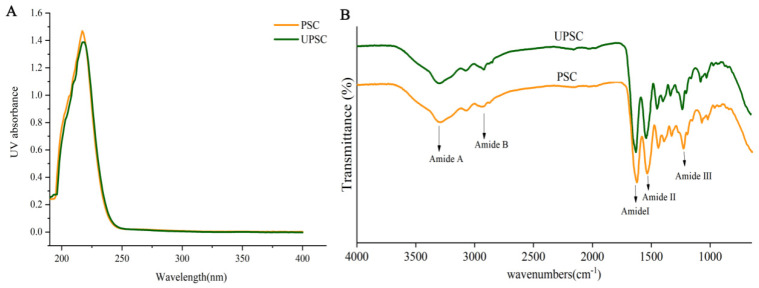
*Bactrian camel* skin collagen samples’ UV absorption spectra (**A**) and Fourier transform infrared spectra (**B**) (PSC, pepsin-solubilized collagen; UPSC, ultrasound−treated pepsin−solubilized collagen).

The PSC and UPSC absorption peaks were observed at approximately 218 nm (Figure 2A). These observations were consistent with the published type I collagen UV absorption spectra from birds, fish, and mammals [25]. Absorption by collagen C = O, –COOH, and CONH_2_ groups are believed to be mainly responsible for these observations [26]. Additionally, the lack of an absorption peak at 280 nm indicated lower contents of tyrosine and phenylalanine in the PSC and UPSC samples [19]. A previous study reported that the lack of an absorption peak at 280 nm in a collagen sample suggests a highly pure sample [27].

### 3.5. Collagen FTIR Spectra

The main absorption peaks of PSC and UPSC were amide A, B, and amide I, II, III (Figure 2B). The amide A peaks of PSC and UPSC were found at 3286 and 3301 cm^−1^, respectively. The absorption characteristic of amide A mainly occurs in the wavenumber range of 3400~3450 cm^−1^, and is usually associated with the stretching vibration of N–H; however, it might shift to a lower frequency if the N-H group forms a hydrogen bond [28]. In PSC and UPSC, the amide B peaks appeared at 2879 and 2922 cm^−1^, respectively, reflecting CH2’s asymmetrical stretch and CH3’s symmetrical stretch [29].

The amide I, II, and III bands’ wave numbers are related to the collagen structure. The range 1600–1700 cm^−1^ usually encompasses the amide I band, which is mainly associated with the stretching vibration of the C = O bond that forms H-bonds between adjacent chains [30]. The amide II band was generally located in the range of 1200 to 1400 cm^−1^, representing N–H bending vibrations and C–N stretching vibrations [31]. The amide III peak represents the stretching vibration of C-N and N-H in-plane bonding, which is related to collagen’s triple helical structure [32]. The amide I, II, and III bands of PSC and UPSC were located at ~1630 cm^−1^, ~1543 cm^−1^, and ~1236 cm^−1^, respectively (Table 2). The FTIR spectra results suggested a stable triple-helical structure in the PSC and UPSC samples and no disruption of collagen’s structure by ultrasound.

### 3.6. Zeta Potential and Particle Size

**Figure 3:**
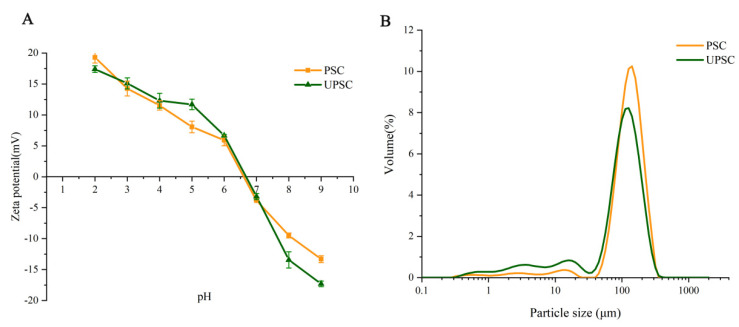
*Bactrian camel* skin collagen samples’ zeta potential (**A**) and particle size (**B**) (PSC, pepsin-solubilized collagen; UPSC, ultrasound-treated pepsin-solubilized collagen).

The surface charge state of protein particles caused by the ionization of amino acid residues was determined using zeta potential analysis [33]. Different pI values of collagen result from variations in the composition and distribution of amino acid residues on the collagen surface [34]. Generally, the pI values of collagens are mainly 6–9 [9]. As shown in Figure 3, the pI values of the PSC and UPSC samples were similar, at 6.6 and 6.7, respectively. As shown in Figure 3B, the average particle size of UPSC was smaller than that of PSC, suggesting that UPSC has better stability. Smaller particle sizes result from the mechanical force generated by ultrasound and the cavitation effect that breaks the aggregated collagen molecules into smaller pieces.

### 3.7. Surface Morphology

**Figure 4:**
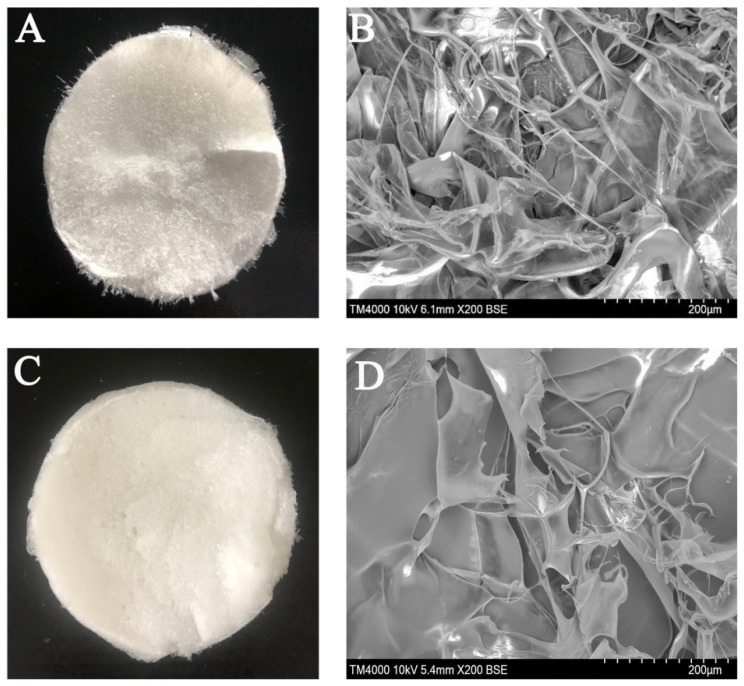
*Bactrian camel* skin collagen as viewed by the naked eye ((**A**): PSC and (**C**): UPSC) and SEM micrograph ((**B**): PSC and (**D**): UPSC) (PSC, pepsin-solubilized collagen; UPSC, ultrasound-treated pepsin-solubilized collagen).

The morphologies of the isolated UPSC and PSC are shown in Figure 4. UPSC looked similar to soft white cotton. However, PSC had a firmer texture compared with UPSC (Figure 4A,C). For the PSC sample, the microscopic structure appeared loose, and many cross-sections with inter-connected fibrillar network pores were observed (Figure 4B). However, the UPSC showed a more multilayered aggregated structure, with a network that was less dominated by fibrils (Figure 4D). Both PSC and UPSC showed the fibrillar properties of collagen, and the length and structure of collagen fibers was affected by ultrasonic treatment [35]. Previous studies have confirmed that the microscopic structure of marine collagen extracted using ultrasound was observed as loose, porous, and homogenous, as compared to conventional method [9,17].

### 3.8. Rheological Properties

**Figure 5:**
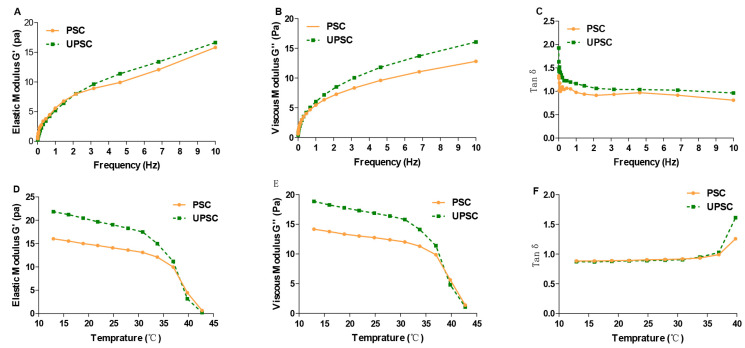
PSC and UPSC samples’ elasticity modulus G′ (**A**,**D**), viscous modulus G″ (**B**,**E**), and loss tangent tanδ (**C**,**F**) in dynamic frequency sweep and temperature sweep tests (PSC, pepsin-solubilized collagen; UPSC, ultrasound-treated pepsin-solubilized collagen).

The elasticity of samples is reflected by the storage modulus (G′), and a sample’s viscosity is reflected by the G″ modulus [36]. Over a range of 0.01 to 10 Hz, we observed increases in the G′ and G″ moduli with increasing frequency of the shear strain (Figure 5A,B), which were similar to the results for grass carp collagen [12]. The G’ modulus of UPSC was consistently larger than that of PSC, which might have been caused by the unwinding of the triple helix structure under the action of ultrasound, ultimately resulting in the formation of a local gel [17]. The G″ modulus of UPSC was also consistently larger than that of PSC, indicating that UPSC has a more stable network structure and better dynamic elasticity. Additionally, the G′ and G″ values of both PSC and UPSC were substantially higher at 0.01 Hz than at 10 Hz, suggesting that at low frequency, the concentration had a more significant effect on the viscoelasticity of the solution system. The Tan δ value crosses the threshold from solid-like to liquid-like behavior (tanδ = 1). The solution system mostly exhibits viscous behavior at tanδ > 1, and elastic behavior at tanδ < 1 [37]. The tanδ value of UPSC was always greater than 1 in the range of 0–10 hz, indicating that the viscosity of UPSC plays a dominant role in the solution system (Figure 5C). However, the tanδ of PSC was less than 1 in the range of 1–10 hz, indicating that the contribution of elasticity to the solution system increased. The main reason for this phenomenon might be that the ultrasound treatment loosened the fibrils and bundles, making the structure less rigid and dense [17].

Changes in G′ and G″ observed during temperature sweeps of *Bactrian camel* skin collagen are presented in Figure 5D,E. The G′ and G″ moduli of PSC and UPSC were decreased at different temperatures. The results showed that collagen of PSC and UPSC all underwent a gelation process at temperature 42.73 °C. This occurs because of the interactions among denatured proteins, which lead to aggregation and gel formation [38]. Below the denaturation temperature, tanδ is less than 1, and the collagen solution displays elastic behavior (Figure 5F). As the temperature approaches the denaturation temperature, increasing viscous behavior occurs in the solution.

### 3.9. Solubility

**Figure 6:**
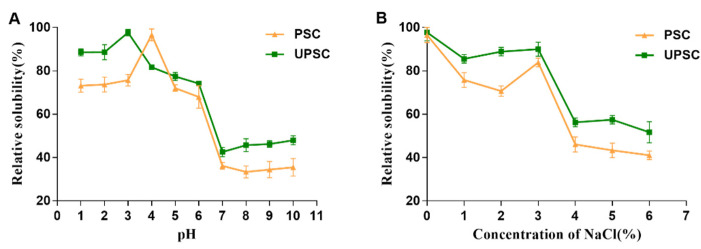
PSC and UPSC relative solubilities (%) at different (**A**) pH values and (**B**) NaCl concentrations (PSC, pepsin-solubilized collagen; UPSC, ultrasound-treated pepsin-solubilized collagen).

Figure 6A shows that the curve of the relative solubility of UPSC was similar to that of PSC. At pH 1–3, UPSC was very soluble, reaching a maximum at pH 3, while the solubility of PSC was the highest at pH 4. At pH 4–7, the solubility of PSC and UPSC decreased significantly, before increasing slightly at pH 7–10, with the lowest solubility at pH 7 and 8, respectively. The slight increase in solubility at higher pH might reflect an increase in the collagen’s net negative charge, which would enhance the repulsion between the chains [39]. UPSC showed consistently higher relative solubility at pH 1–10 compared with that of PSC. This might reflect UPSC’s enhanced degree of cross-linking or stronger bonds.

UPSC and PSC showed high solubility up to 3% (*w*/*v*) NaCl (Figure 6B). Collagen solubility in both groups decreased significantly at 4% (*w*/*v*) NaCl, followed by a gradual decline in higher salt concentrations. At high concentrations, salt ions disturb the hydration shell on the collagen’s surface to expose hydrophobic sites. This exposure increases the likelihood of hydrophobic interactions, resulting in aggregation and precipitation of collagen [40].

## 4. Conclusions

PSC and UPSC were successfully extracted and characterized from the Bactrian camel. Ultrasound facilitated the pepsin-mediated extraction of collagen and promoted collagen yield. The FTIR spectra, UV absorption spectra, electrophoretic mobility pattern, and amino acid composition demonstrated that the extracted collagen is type I collagen and retains its structure. UPSC from *Bactrian camel* skin could be used as a novel source and will be beneficial in promoting the commercialization of *Bactrian camel* skin.

## Figures and Tables

**Table 1:** *Bactrian camel* skin PSC and UPSC amino acid compositions.

Amino Acids	Content (% of Total Amino Acid)	
	PSC	UPSC
Aspartic acid	5.31	5.35
Threonine	1.99	2.00
Serine	3.17	3.22
Glutamine	9.40	9.45
Glycine	22.70	22.68
Proline	11.61	11.31
Alanine	8.39	8.39
Valine	2.37	2.37
Methionine	0.66	0.65
Isoleucine	1.12	1.13
Leucine	2.67	2.83
Tyrosine	0.73	0.72
Phenylalanine	2.06	1.95
Histidine	0.66	0.65
Lysine	3.61	3.49
Cysteine	0.46	0.33
Arginine	7.58	7.77
Hydroxyproline	7.92	8.25
Imino acid	19.53	19.56

**Table 2:** PSC and UPSC FTIR spectra peak locations and assignments.

Region	Peak Wavenumber/cm^−1^		Assignment
	PSC	UPSC	
**Amide A**	3286	3301	N-H stretch, coupled with hydrogen bond formation
Amide B	2879	2922	CH2 asymmetrical stretch CH3 symmetrical stretch
Amide I	1630	1630	C = O stretch/hydrogen bond coupled with COO-
Amide II	1543	1544	N–H bend coupled with C–N stretch
	1449	1450	CH2 bend
	1400	1400	COO-symmetrical stretch
	1337	1337	CH2 vibration
Amide III	1236	1236	N-H bend coupled with C-N stretch
	1081	1081	C-O stretch
	655	656	Skeletal stretch

## Data Availability

Data are contained within the article.
